# Improvement of Emotional Response to Negative Stimulations With Moderate-Intensity Physical Exercise

**DOI:** 10.3389/fpsyg.2021.656598

**Published:** 2021-07-01

**Authors:** Zhengji Long, Guangyuan Liu, Zhangyan Xiao, Pengfei Gao

**Affiliations:** ^1^College of Electronic and Information Engineering, Southwest University, Chongqing, China; ^2^Chongqing Key Laboratory of Nonlinear Circuits and Intelligent Information Processing, Chongqing, China

**Keywords:** physical exercise, EEG asymmetry, moderate intensity, emotion regulation, negative emotion

## Abstract

It is widely accepted that physical exercises (PEs) not only are good for fitness but also contribute to mental health and well-being. The positive influence of PEs on emotion has become a topic of much excitement. However, a quantitative study is required to discuss the effect of short-term moderate-intensity PE on the emotional response by using electroencephalogram (EEG) asymmetry. The experiments, including 20-min moderate-intensity cycling and EEG data acquisition with picture-induced emotion assessment protocol, were designed in this paper. The experiment procedure consists of two emotion assessment sessions, each of which contains 24 pictures. About 80 participants were randomly allocated into the exercise group and the control group. Participants in the exercise group were instructed to have a 20-min moderate-intensity cycling after the first assessment session, then rested until their heart rates recovered to baselines and their emotional states were assessed again in the second session. The control group only had a 20-min break without the cycling exercise between the two sessions. It was observed that, in the control group, the EEG asymmetry had no significant difference in these two assessment sessions for both positive and negative stimulations. However, in the exercise group, the difference of the EEG asymmetry before and after PE was significant only in response to negative stimulations. Further, the in-depth analysis of EEG asymmetry index changes of individual participants shows that the short-term moderate-intensity PE has a positive impact in response to negative stimulations. The proposed experiments show that the negative emotional experience can be reduced by the moderate-intensity PE and support the hypothesis that the moderate-intensity PE is good at improving emotional response to negative stimulations. This study provides the evidence of positive effects of PE in the domain of emotion regulation with experimental data.

## Introduction

Emotion experience is a complex procedure of neurobiological activity related to sensation, consciousness, and behavior that reflects the personal significance of a thing, event, or state of affairs. One of the key areas of emotion study is the interaction between the behavior of a person and the emotional states of a person. Emotional experience is ubiquitous in nature, and how the negative and positive emotion states change plays an essential role in promoting career success of a person or facilitating creative problem solving (Isen et al., 1987; Lyubomirsky et al., 2005; Boehm and Lyubomirsky, 2008). Understanding emotion change processes is a primary objective for supporting emotional well-being, which helps improve mental healthcare. With the rapid development of computer and information technology, the affective computing can be used to understand the emotion and behavior of a person and analyze how the emotion of the person changes. This has gained intense attention in biomedical engineering, computer science, and psychophysiology in the last few decades. Change of emotion, also known as mood change, refers to how the emotion or the intensity level of mood changes in response to internal or external affairs. For example, sometimes, the mood of an individual will swing from feeling confident and joy to feeling worried and depressed in a short period of time, and vice versa. Another example is that the depressing or happy feeling can last longer at a higher intensity level to a great extent. More specifically, the study of emotion change is about which emotions we have, when, how, and to what degree we have them, also known as emotion regulation (Gross, 1998, 1999).

There have been many psychology studies on various emotion regulation strategies to affect the changes of emotion (Gross, 2002, 2015; John and Gross, 2004; Quoidbach et al., 2015). In particular, emotion regulation is essential in the changes of negative emotion, which is useful in human mental well-being. It is well-known that physical exercise (PE) is not only good for fitness but also improves mental health (Penedo and Dahn, 2005; Belcher et al., 2021; Smith and Merwin, 2021). It is also claimed that PE affects the emotion of a person, in both clinical and non-clinical populations. For example, for negative emotion, acute aerobic exercise helps overcome emotion regulation deficits and hastens emotional recovery from a subsequent stressor (Bernstein and McNally, 2017a,b). The effects of different intensities and rest period during resistance exercise on anxiety and negative affect were examined (Bibeau et al., 2010). The benefits of aerobic exercise on the neural efficiency in responding to sad emotion-eliciting cues were found (Hwang et al., 2019). Frequent PE may improve the efficiency of controlling negative emotions in women (Ligeza et al., 2019). After acute aerobic exercise, participants showed a decline in state anxiety (Petruzzello and Landers, 1994; Cox et al., 2004). Exercise can improve depressive symptoms in people with a diagnosis of depression (Strohle, 2009; Rimer et al., 2012; Schuch et al., 2016a,b). On the other hand, for the positive emotion, subjects showed increased positive affect during exercise (Boutcher et al., 1997). Research consistently showed that PE is associated with pleasant changes and positive affective valence for most individuals (Ekkekakis et al., 2000, 2011). Increases in dispositional mindfulness were moderately correlated with improvements in mental health, and dispositional mindfulness can be increased through regular aerobic exercise (Mothes et al., 2014). Most studies suggest that PE helps to reduce state anxiety, depression, and tension. PE also increases the feelings of vigor and happiness, and it improves various emotional states. The research studies on PE-induced emotion changes mainly focus on how to regulate the existing negative emotions for those people with mental or emotional problems, for example, anxiety and depression. PE is an effective method for mental disorder treatment and also a kind of posterior measure of negative emotion. There are few reports regarding the effects of PE on the emotional response to the subsequent stimulations.

However, due to the complexity of the change of emotion, it is still not clear if PE has similar effects on both positive and negative emotions. There is a lack of quantitative study and neurobiology data to investigate the different impacts of PE on positive and negative emotions, as people experience a wide range of emotions after PE. Research in this field should concern not only the regulation of existing emotional states but also the in-depth awareness of how PE can be used to improve negative emotions and/or the emotional responses to negative events. There are still some open questions to be addressed. It is still a lack of in-depth understanding with evidence of whether PE can help to prompt positive emotions or reduce the changes toward negative emotions. In other words, the hypothesis to be investigated in this paper is whether the positive emotional experience will be enhanced, while the negative emotion is reduced after some PE.

Furthermore, some studies have found that PE can work as an emotion-induction event that generates emotions. In particular, when the intensity of the exercise is at an appropriate level, positive emotion can be induced. The following question to be addressed is how the negative emotion is affected by PE. Is the reduced level of negative emotion caused by the neutralization of the positive emotion induced in the PE? This is particularly interesting for short-term moderate-intensity PEs, as the recovery period after a short-term moderate-intensity PE is relatively short. Will the positive emotion last longer and lead to the reduction of negative emotion? After the physiological indicators are restored, will the role of emotion regulation still exist? The human emotion research community has not reached a consensus regarding the psychological and physiological mechanisms behind the emotion changes, which inspire this study about the effects of moderate-intensity PEs on the emotional response to negative stimulation.

Although significant progress has been made in understanding emotion and the emotion regulation process in the psychological community, neurobiology evidence of emotion changes is not enough, due to the complex mechanisms underlying human emotions. Therefore, there is a need to provide qualitative neurobiology data to better recognize the emotion change after PE. One typical neurobiology data widely used for emotion analysis is the electroencephalogram (EEG). Since Davidson found that the asymmetry of activation in the anterior part of the brain is closely related to emotion (Davidson et al., 1990), and the asymmetry of frontal EEG signal has been widely used as a key index of human emotional states in many studies. It has been shown that the greater activity of the left frontal is related to the approach system and positive (pleasant) emotion. On the other hand, the greater activity in the relative right frontal is involved avoidance/withdrawal system, thus related to negative (unpleasant) emotion (Davidson, 1998, 2000, 2004). The asymmetry of the EEG activity in the prefrontal region has been discussed as a predictor, outcome, moderator, and mediator of emotion in a large number of studies (Hall et al., 2000, 2010; Coan and Allen, 2004; Reznik and Allen, 2018). In summary, the results show that individuals with greater frontal activity on the left are relatively in a positive emotional state and can better regulate negative emotions.

In the study, the advanced EEG signal processing technology is used to investigate the hypothesis that moderate-intensity PE can improve emotion experience for both negative and positive stimulations. The moderate-intensity PE is selected owing to its population in the daily exercise. The study may provide further evidence on the positive effects of PE in emotion regulation and suggestions for maintaining mental health and emotional well-being. This paper is organized as follows: section Materials and Methods presents the proposed experiment procedure, the materials used in the experiments, and the signal processing methods. Section Data Analysis and Results presents the data analysis and the experimental results, followed by the discussion and conclusions in section Discussion and Conclusion.

## Materials and Methods

### Experiment Method

**Figure 1:**
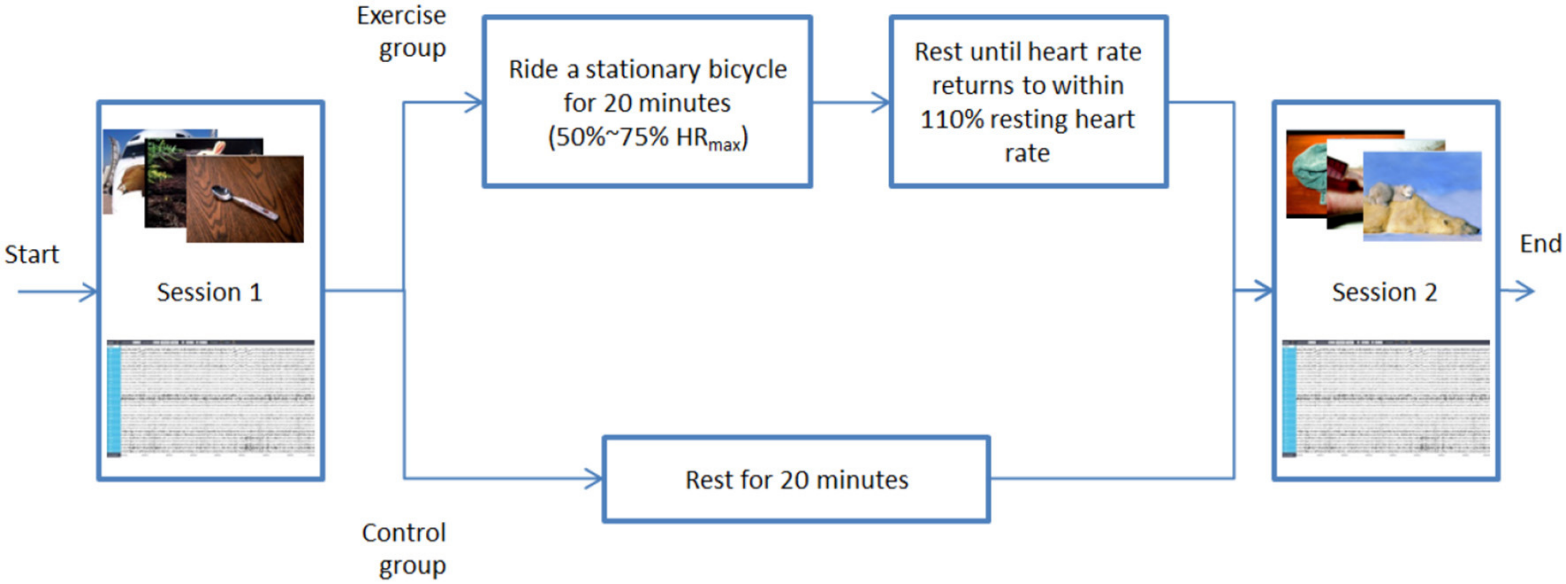
The procedure of the proposed experiment.

Total 48 emotional pictures are carefully selected as stimulation for evoking emotion. Both negative and positive emotional pictures are included in this experiment, as shown in Figure 1. The experiment consists of two assessment sessions, and between the two assessment sessions, either a 20-min cycling PE session for the exercise group or a 20-min rest session for the control group is included. In each assessment session, a sequence of emotional pictures with similar valance is presented to the participants. The EEG signals are collected to determine the emotional state of the participants. After the first session, the participants in the exercise group take 20 min of moderate-intensity exercise followed by a recovery period to allow the participants to take a rest until their heart rates recovery to normal status. The control group does not take the exercise and provides a benchmark to evaluate the difference caused by the moderate PE. Rather than taking a 20-min exercise and recovery period between the two sessions, the participants in the control group just take a 20-min rest. All other procedures are precisely the same as those in the exercise group. The detailed procedures of the experiment will be presented later.

### Participants

The participants (*N* = 80) were healthy college students aged 18–25 years who were recruited at Southwest University in China. All potential participants were initially assessed by the results of the international physical activity questionnaire (IPAQ) (Hagstromer et al., 2006). The selected participants consisted of 40 male students (age 20.18 ± 1.43, height 174.08 ± 5.58 cm, and weight 63.78 ± 8.70 kg) and 40 female students (age 19.9 ± 1.43, height 161.28 ± 5.42 cm, and weight 52.33 ± 5.87 kg). All participants were right-handed, with normal vision and color perception, body mass index (BMI) < 25, and no mental, neurological, cardiovascular disease, or physical disability. All participants must have no exercise habits. The criteria of “exercise habits” are defined as that the frequency of taking moderate exercise at least three times a week for a period of more than 1 year, and each exercise lasts more than 30 min. They were divided randomly but equally in gender into the exercise group and control group. There were 20 male and 20 female subjects in each group. The basic demographic information is the same in the two groups. The participants were asked not to exercise 24 h before the experiment and to wear comfortable clothes and shoes. This study was conducted based on the ethical principles of the Declaration of Helsinki regarding human experimentation (WMA., 2013) and was approved by the local Review Board for Human Participant Research. Each participant signed informed consent before the experiment.

### Pictures

Sixteen positive, negative, and neutral pictures, respectively, were selected from the International Affective Picture System (IAPS) (Lang et al., 2008) according to valance and arousal. Each session contains 24 pictures consisting of eight pictures for positive, neutral, and negative emotional states. These pictures are used to arouse emotions. Table 1 shows the statistics of the selected pictures and the *p-*value of the significance test for the valence and arousal of each kind of picture in the two sessions. The valance and arousal have no significant difference for negative, neutral, and positive pictures in the two sessions, respectively.

**Table 1:** Pictures for emotion induction.

**Picture type**	**Dimension**	**Session 1**		**Session 2**		***p***
		**Mean**	**SD**	**Mean**	**SD**	
Negative	Valence	2.88	0.97	2.87	0.96	0.9962
	Arousal	5.8	1.51	5.76	0.91	0.9546
Neutral	Valence	4.99	0.04	4.99	0.04	0.6814
	Arousal	3.68	1.11	3.26	1.17	0.5113
Positive	Valence	6.96	1.02	6.95	1.01	0.991
	Arousal	4.3	0.83	4.28	0.85	0.9496

### Mode and Duration of Exercise

**Figure 2:**
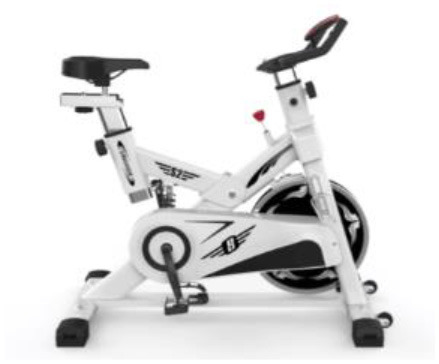
Stationary bicycle used in the experiment.

The exercise used in the experiment is 20-min cycling on a stationary bicycle shown in Figure 2 (Snode S2, http://www.sinuode.cn/). To ensure that the exercise is at the level of moderate intensity, the heart rate of the participant is monitored and kept at 50–75% of the maximum heart rate (HR_max_) by adjusting the resistance of the flywheel. The HR_max_ is estimated by the following formula (Tanaka et al., 2001).

(1) $$\begin{array}{r} HR_{max}=208-0.7\times age \end{array}$$

The duration of the exercise is started counting when the heart rate of the participant reaches 50% *HR*_*max*_.

### Electroencephalogram Recording

**Figure 3:**
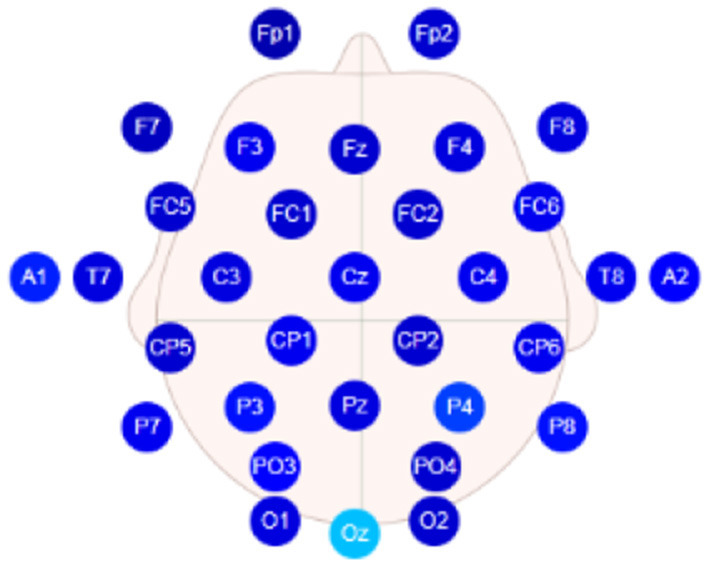
Electrode placement for Neuracle system.

Continuous EEG was recorded from 32 scalp electrodes placed according to the international 10–20 system using the wireless EEG recording equipment provided by Neuracle (NEUSEN W, 32 channels, http://www.neuracle.cn/productinfo/148706.html). The selected positions for the scalp electrode placement are shown in Figure 3, and the sampling rate is 250 Hz.

### Procedures

During the experiments, the participants wore comfortable clothes and shoes and were informed of the experimental process and precautions before starting the experiments. After signing the informed consent form, the participants wore EEG caps. Then EEG data were collected for every participant in these two parallel groups. The detailed procedure is presented as follows:

#### Step 1 (First Assessment Session)

**Figure 4:**
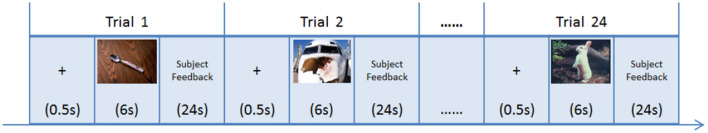
The procedure of the assessment session. Same for both the first assessment session and second assessment session.

The experiments started with the first assessment session, where 24 pictures, including eight positive, eight negative, and eight neutral, were presented to the subjects in a random sequence. Figure 4 shows a typical example of the picture sequence in one trial. Before presenting each picture to the participant, a fixation point picture was first displayed for 0.5 s to remind the subjects to pay attention to the screen. Then a picture was presented for 6 s, followed by a 24 s gap to fill in the 9-level valance scale. The reason for setting a longer time after the picture presentation is to avoid the impacts of emotional reaction induced by the previous picture on the successive one. The presentation procedure based on 0.5–6–24 s picture was repeated 24 times until all the pictures are presented.

#### Step 2 (Exercise/Rest)

The 20-min cycling was conducted. Before the experiment, the subjects in the exercise group wore a sports watch (HUAWEI WATCH GT) and were measured the resting heart rate, then started to cycle with a stationary bicycle (Figure 2). When the real-time heart rate of the subjects reached 50% of their maximum heart rate, the timing began, and then the heart rate was maintained within the medium intensity range (50–75% *HR*_*max*_) for 20 min. The subjects would be asked about the subjective exercise intensity feeling, and the heart rate was recorded every 2 min. After completing the 20-min cycling, all participants in the exercise group had a rest of about 20 min until their heart rates settled down to no more than 10% of the resting heart rates before the experiment. Then, the second session of the experiment started. On the other hand, the subjects in the control group only rest for 20 min without exercise in this step.

#### Step 3 (Second Assessment Session)

In the second assessment session, other 24 pictures are conducted. The procedures of presenting the picture in the second assessment session are the same as those of the first assessment session except the used different pictures. The same procedure at this step is used for both the exercise group and the control group.

### Electroencephalogram Asymmetry Index

It is well-known that the asymmetric frontal alpha of hemispheres is related to emotional states. The relatively greater activities on the right frontal portion of the brain are associated with negative emotions, for example, sadness, fear, and disgust. In contrast, the relatively greater activities on the left frontal lobe are related to positive emotions, for example, joy and happiness. Furthermore, there is a significantly negative correlation between brain activities and the power spectral density (PSD) of the alpha band signal. Similar to related work in literature (Hall et al., 2010; Papousek et al., 2012; Koller-Schlaud et al., 2020; Zhang et al., 2020), electrodes F3 (left hemisphere) and F4 (right hemisphere) were selected as the main data source for analysis. The asymmetry index of frontal lobes is defined as:

(2) $$\begin{array}{r} Asy\left[k,i,j\right]=\frac{PSD_{r}\left[k,i,j\right]-PSD_{l}\left[k,i,j\right]}{PSD_{r}\left[k,i,j\right]+PSD_{l}\left[k,i,j\right]} \end{array}$$

where *PSD*_*l*_ and *PSD*_*r*_ are the PSD of the alpha band signal. *PSD*_*l*_ and *PSD*_*r*_ were recorded from electrode F3 in the left hemisphere and from electrode F4 in the right hemisphere, respectively. Indices [*k,i,j*] represent the *k*-th subject, *i*-th picture in the picture stimulations, and *j*-th segment of the EEG data. It can be seen from the definition of asymmetry index that a positive asymmetry index (*Asy* > 0) corresponds to the relatively greater left frontal activity (*PSD*_*r*_ > *PSD*_*l*_), whereas a negative asymmetry index (*Asy* < 0) represents relatively more significant right frontal activity.

**Figure 5:**
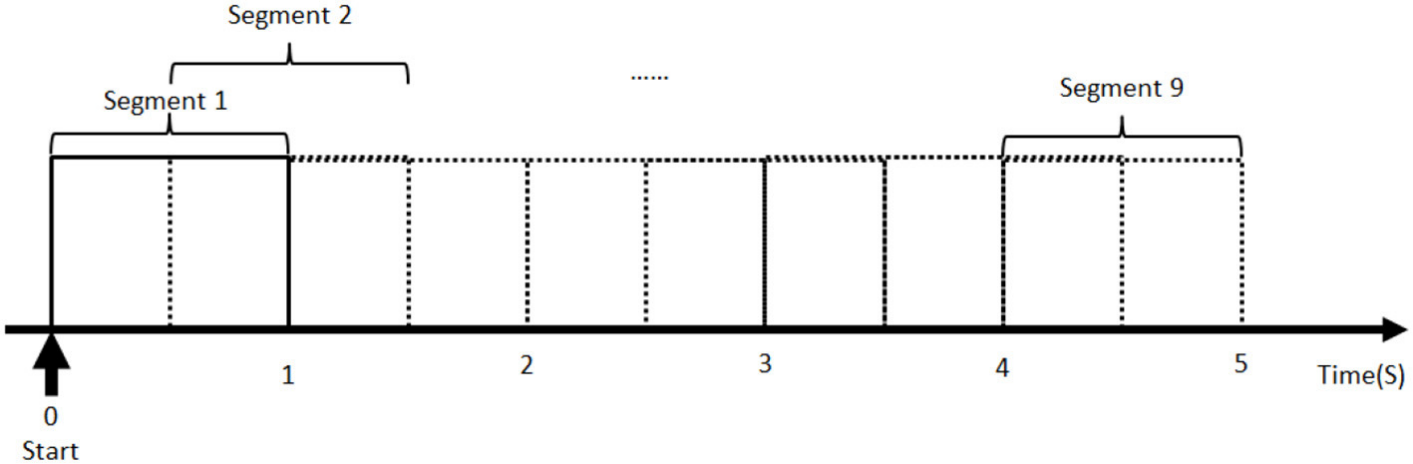
Data segmentation of each picture stimulation trial.

Data collected from subjects in two different groups were analyzed. Data preprocessing showed that the EEG data of 37 subjects in the exercise group are valid. So are the 33 subjects in the control group, as some subjects did not complete the experiment or the marker information of the data was not complete. A 0.5-Hz high-pass filter is used to process all the valid data for removing direct current drift (Liu et al., 2018). Since the duration of emotion induced by pictures may be short, the filtered data were segmented into a sequence of a 1-s moving window to meet the requirement of data length for the power spectrum estimation (Jatupaiboon et al., 2013). Each window contains 250 data because the sampling rate is 250 Hz. The overlap ratio of successive data windows is 50% (i.e., 125 data for a half-second) (as shown in Figure 5). Although every picture was displayed for 6 s, only the first 5-s data were adopted for the frontal activity analysis. This is because the data in the last second contain more noise, and the emotional duration aroused by the picture stimulation is generally short. As a result, nine segments of the EEG data were obtained for each frontal lobe in every trial. The power spectrum of each EEG channel (F3 and F4) was estimated by the Welch method (Jwo et al., 2021). Here, Hamming window with the length of 50 and overlap of 50% was adopted. The EEG signals observed in the scalp were divided into specific ranges, namely the alpha (8–13 Hz), beta (13–30 Hz), gamma (>30 Hz), delta (1–4 Hz), and theta (4–7 Hz) bands. The beginning and the end of the bands can be set with a slight difference (Alarcao and Fonseca, 2019). PSD of the alpha frequency band (8–13 Hz) was retrieved for each segment of the left frontal lobe (F3) and right frontal lobe (F4) data, followed by the calculation of the asymmetry index *Asy* in Equation (2). All the aforementioned procedures were performed in the EEGLAB toolbox (Delorme and Makeig, 2004) and MATLAB (MathWorks, Natick, MA, USA).

## Data Analysis and Results

With all the asymmetry indices collected, statistical comparisons of all segments were made to evaluate the effect of short-term moderate-intensity PE on emotion regulation. For negative stimulation in the exercise group, the number of EEG segments with *Asy* < 0 in the post-exercise session was less than that in the pre-exercise session. We also define the mean value of the maximum asymmetry index from all the trials and its minimum $Mean\left(A_{sy}^{\min}\right)$ as follows.

(3) $$Mean\left(A_{sy}^{max}\right)=\frac{\sum_{k,i}\left(\max_{j}\left(Asy\left[k,i,j\right]\right)\right)}{8n}$$

(4) $$Mean\left(A_{sy}^{min}\right)=\frac{\sum_{k,i}\left(\min_{j}\left(Asy\left[k,i,j\right]\right)\right)}{8n}$$

where *n* is the number of subjects, 37 in the exercise group, and 33 in the control group, respectively. Indices [*k,i,j*] are the same as those defined in Equation (2).

Statistical results of segments are shown in Table 2. For the exercise group, session 1 and session 2 correspond to pre-exercise and post-exercise, respectively. In Table 2A, compared with the pre-exercise session (session 1), $Mean\left(A_{sy}^{\max}\right)$ increased and the number of segments with *Asy* < 0 in the post-exercise session (session 2) decreased. These were highlighted in bold faces. The decreased number of EEG segments with *Asy* < 0 indicates that the negative emotion duration in post-exercise sessions is less than that in pre-exercise sessions. Meanwhile, the mean value of the maximum index increases, which implies that relatively greater activities in the left frontal are enhanced as expected. On the other hand, in the control group, the number of EEG segments with *Asy* < 0 in session 2 was slightly more than that in session 1, and there was less change in $Mean\left(A_{sy}^{\max}\right)$ and $Mean\left(A_{sy}^{\min}\right)$.

**Table 2:** Statistical results of segments.

**(A) Negative stimulations**							
**Group**	**Total segments**	**Session 1**			**Session 2**		
		**Segments Asy < 0**	$Mean\left(A_{sy}^{min}\right)$	$Mean\left(A_{sy}^{max}\right)$	**Segments Asy < 0**	$Mean\left(A_{sy}^{min}\right)$	$Mean\left(A_{sy}^{max}\right)$
Exercise	2,664	**1,213**	−0.223	**0.255**	**1122**	−0.217	**0.270**
Control	2,376	1215	−0.245	0.237	1226	−0.254	0.234
**(B) Positive stimulations**							
**Group**	**Total segments**	**Session 1**			**Session 2**		
		**Segments Asy > 0**	$Mean\left(A_{sy}^{min}\right)$	$Mean\left(A_{sy}^{max}\right)$	**Segments Asy > 0**	$Mean\left(A_{sy}^{min}\right)$	$Mean\left(A_{sy}^{max}\right)$
Exercise	2,664	1,451	−0.227	0.263	1,492	−0.221	0.263
Control	2,376	1,152	−0.249	0.234	1,121	−0.240	0.225

In Table 2B, for positive stimulation in the exercise group, the number of segments with *Asy* > 0 in the post-exercise session increased slightly, and there were no significant changes in $Mean\left(A_{sy}^{\max}\right)$ and $Mean\left(A_{sy}^{\min}\right)$. In the control group, the number of segments with *Asy* > 0 in session 2 decreased slightly, and the range of *Asy* changed to negative direction slightly.

**Figure 6:**
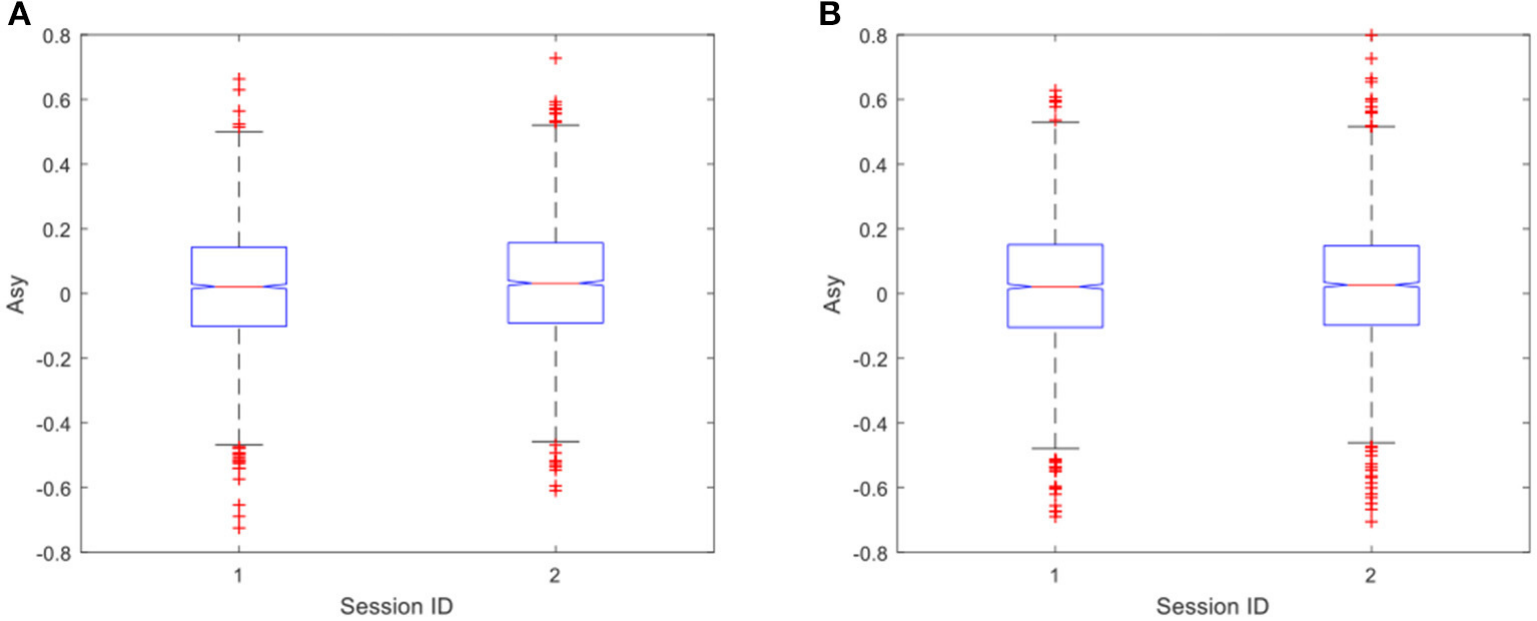
**(A)** Difference in *Asy* for negative stimulations (*p* = 0.0254) in the exercise group; **(B)** Difference in *Asy* for positive stimulations (*p* = 0.3361) in the exercise group. Here, session ID = 1 is the pre-exercise session and session ID = 2 is the post-exercise session.

In the data analysis, the ANOVA is adopted to identify any statistically significant differences between the values of *Asy* in pre-exercise and post-exercise sessions. In the exercise group, the results of ANOVA showed that, for negative stimulations, there was a significant difference in *Asy* between the pre-exercise and post-exercise sessions with *p* = 0.0254, as shown in Figure 6A. However, it is interesting to note that there was no significant difference in *Asy* for positive stimulations, where the value of *p* is just 0.3361, as shown in Figure 6B.

**Figure 7:**
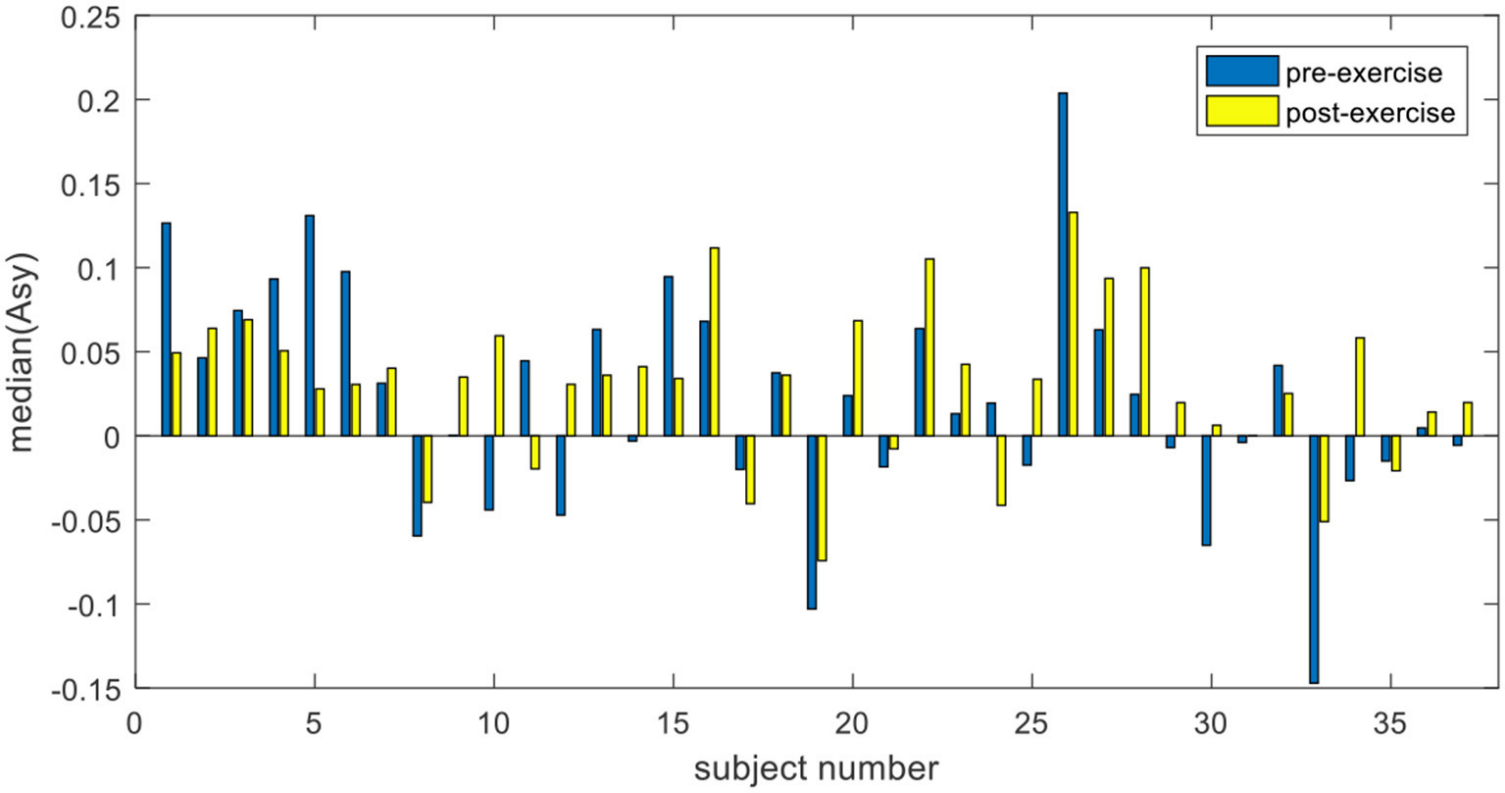
Comparison of Asy. median in the exercise group.

**Figure 8:**
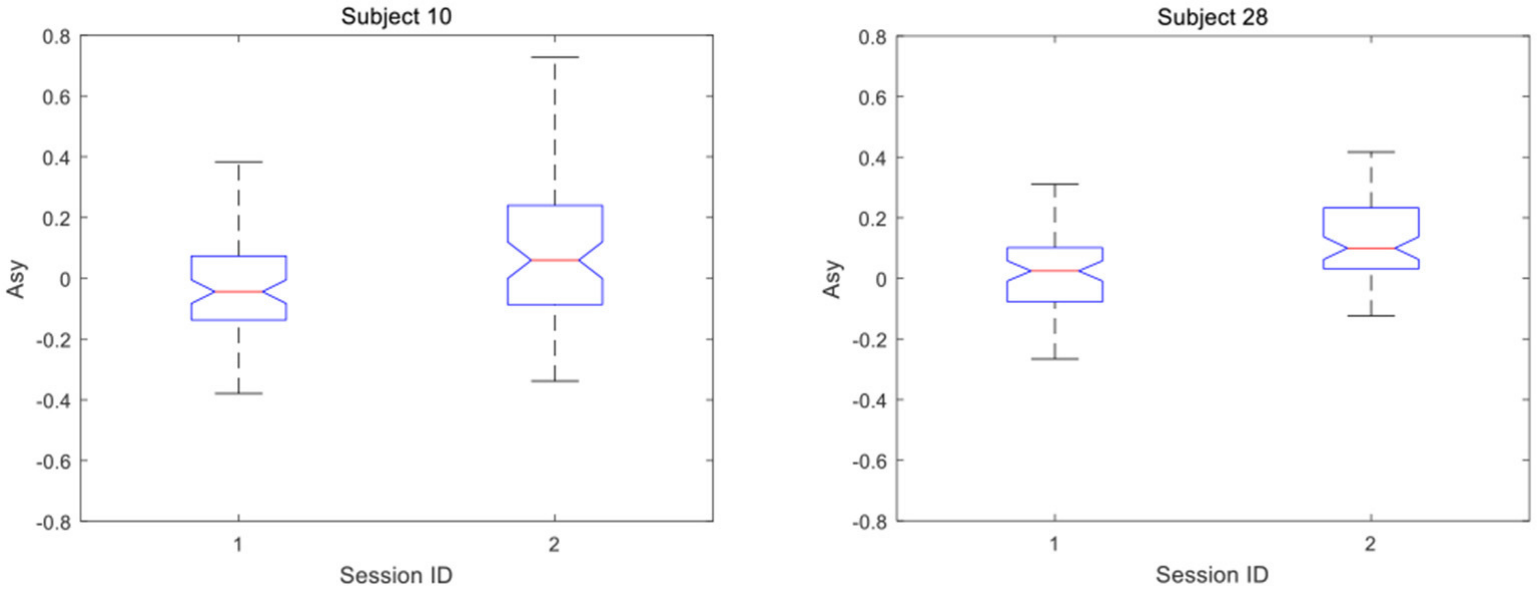
Asymmetry index of subjects increases positively.

Furthermore, analysis of EEG data of an individual shows that the significant difference in the exercise group was due to the positive increase of *Asy*. For each subject in the exercise group, under the condition of negative stimulations, 72 asymmetry indices in the pre-exercise session are combined into one group, and 72 asymmetry indices in post-exercise session are combined into another group. The median values of *Asy* before and after exercise were compared. The trend can be seen clearly from Figure 7 that 23 out of the 37 subjects in the exercise group changed positively in the asymmetry index before and after the exercise. Among them, the asymmetry index moved significantly toward the right, either changed from negative to positive or from a lower positive value to a higher positive value. In Figure 8, the details of the two subjects are presented, which can be seen more clearly.

**Figure 9:**
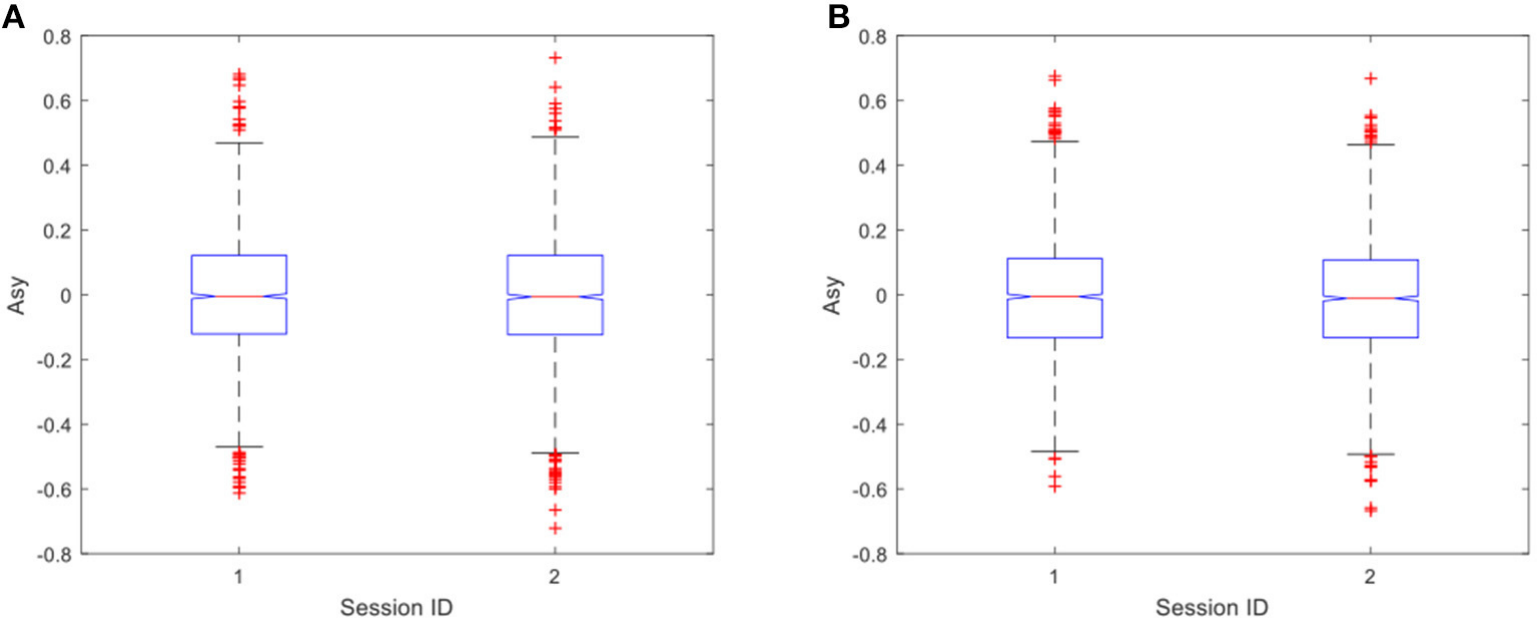
**(A)** Difference in asymmetry index for negative stimulations (*p* = 0.627) in the control group. **(B)** Difference in asymmetry index for positive stimulations (*p* = 0.4666) in the control group.

The same test was also carried out for Asy of the control group. The results showed no significant difference in Asy between the two sessions under both positive and negative stimulations, as shown in Figures 9A,B. The values of *p* were 0.627 and 0.4666, respectively, which shows that PE has no significant impact on positive emotional experience in both two groups.

## Discussion and Conclusion

As shown in the EEG signal analysis, the EEG asymmetries in the exercise group are of significant difference before and after the 20-min cycling exercise, and the mean of median changes from 0.0212 in the pre-exercise session to 0.0318 in the post-exercise session. This is true in response to emotion induced by negative pictures (as shown in Figure 6A, *p* = 0.0254). However, a significant difference in EEG asymmetry in the control group is not observed (as shown in Figure 9A, *p* = 0.627). This clearly demonstrates that the short-term moderate-intensity PE positively impacts emotional experience in response to the subsequent negative stimulation. This study provides evidence for preventing negative emotions by using PE. Individual EEG asymmetry result (as shown in Figure 7) shows that 23 of the 37 participants in the exercise group have a positive difference in response to the negative emotions. It was observed that the asymmetry index *Asy* moved toward the right, either changed from negative to positive or from a lower positive value to a higher one. This is manifested explicitly as the reduction of asymmetrical right lateralization in the process of negative emotional experience and the positive change of *Asy*. However, we only observed this positive change among some participants in the exercises group. We infer that this may be related to the mode, intensity, and duration of exercise, and some studies have also shown that these may have impacts on the emotion after exercise (Woo et al., 2009, 2010). The effects of different exercise modes, intensity, and duration on the positive change will be discussed in our future work.

It can be found that the reduced level of negative emotion is not caused by the neutralization of the positive emotion induced in the PE. As suggested by some studies in the literature (Petruzzello and Tate, 1997; Hall et al., 2007, 2010; Bibeau et al., 2010; Fumoto et al., 2010), the positive emotional state after PE is produced in the recovery period after exercise. In this study, the exercise load is of moderate intensity and lasts for 20 min. After the recovery period of 20 min, the heart rate had returned to a normal state in the second session. We believe that the positive emotions generated by exercise no longer exist, which is also supported by the later analysis of positive stimulation. However, since the positive effect to negative stimulation still exists after the recovery period, the neutralization of the positive emotion from PE is not the reason for the reduction of negative emotion. The duration of this positive effect and its relationship with the cumulative effect of regular PE will be our further work.

However, compared to the significant impacts of PE on emotion regulation in response to the negative stimulations, it is interesting to observe that there was no significant difference in the positive stimulations, in both the exercise group (as shown in Figure 6B, *p* = 0.3361) and the control group (as shown in Figure 9B, *p* = 0.4666). In the aspect of enhancing positive emotional experience, no relevant evidence was found in the experiment, which is consistent with another study (Crabbe et al., 2007). As a result, the significant impacts of PE on the positive emotions were not observed in our experiment.

In this study, there are some limitations. In the experiment, the intensity of the exercise is controlled by limiting the heart rate to 50–75% of the maximum heart rate and the duration to 20 min. The subjective feelings of the participants are not fully considered. According to the feedback information of the subjects during the experiment, although the subjective self-reports of the pictures are consistent with the IAPS score (positive or negative), there is no significant difference between the two sessions, which is inconsistent with the results of data analysis. Besides, we do not consider the subjective evaluation of arousal.

In summary, our experimental results clearly show that moderate-intensity PE can reduce the negative emotional experiences of individuals. The asymmetry index in post-exercise changed from negative to positive or increased to a higher value in response to the negative stimulation compared with pre-exercise. It indicates that the short-term moderate-intensity PE has a positive impact on the emotional response of people, in particular, to the negative stimulations. It provides quantified evidence for the hypothesis that emotion experience can be affected by PE, in particular, in the regulation of negative-picture-induced emotions.

## Data Availability Statement

The raw data supporting the conclusions of this article will be made available by the authors, without undue reservation.

## Ethics Statement

The studies involving human participants were reviewed and approved by the ethics committee of Southwest University. The patients/participants provided their written informed consent to participate in this study.

## Author Contributions

GL provided the ideas and reviewed the manuscript. ZL, ZX and PG designed the experiment and conducted the experiments to collect the data. ZL analyzed the data and wrote the manuscript.

## Conflict of Interest

The authors declare that the research was conducted in the absence of any commercial or financial relationships that could be construed as a potential conflict of interest.
